# Hemibody Irradiation for Bone Metastases: A Systematic Review and Meta-Analysis

**DOI:** 10.7759/cureus.51925

**Published:** 2024-01-09

**Authors:** Lawrence Berk, Charles Scarantino, Steven Finkelstein, Mitchell Finkelstein

**Affiliations:** 1 Radiation Oncology, Tampa Oncology and Proton, Winter Haven, USA; 2 Radiation Oncology, University of North Carolina, Raleigh, USA; 3 Radiation Oncology, Florida Cancer Affiliates, Panama City, USA; 4 Radiation Oncology, Barrett, The Honors College at Arizona State University, Tempe, USA

**Keywords:** meta-analysis, cancer treatment toxicity, clinical efficacy, palliative radiation therapy, bone-metastases

## Abstract

Hemibody irradiation (HBI) is a radiation therapy technique that involves treating one-half of the patient’s skeletal system in a single radiation field. It is mostly given as upper hemibody irradiation (UHBI), lower hemibody irradiation (LHBI), or sequential UHBI and LHBI. It is used to treat extensive bone metastases from solid tumors. It was primarily utilized in the 1980s and 1990s and has since fallen out of favor. However, it is a potentially cost-effective treatment for widespread bone metastases. To determine its efficacy, we performed a meta-analysis of all available published articles on the efficacy of HBI to relieve pain from bone metastases. Twenty-seven articles involving 1318 patients were identified and analyzed. Our findings show that 80% of the patients had complete or partial pain relief and 29% had complete pain relief. The trials were of poor quality, but the results showed minimal heterogeneity in the response rates. These response rates are consistent with those seen with focal irradiation of bone metastases and for radionuclide treatment of bone metastases. The toxicity of the treatments decreased when delivered with modern treatment techniques. In light of this, we propose that this technique warrants re-evaluation with modern treatment methods.

## Introduction and background

Bone metastases are common in cancer patients. A 2020 study of the SEER database from 2010 to 2013 reported that 5% of patients have bone metastases at presentation, with the highest prevalence in breast and prostate cancer patients [1]. A 2023 study of the Premier Healthcare Database from January 2015 to December 2020 found that the primary sites among patients with bone metastases were the lungs (25%), prostate (19%), and breasts (19%) [2]. A 2015 study based on the Oncology Services Comprehensive Electronic Records database found that breast cancer patients experience a significant symptom burden from bone metastases, including pain, fatigue, sleeping problems, and neuropathic symptoms [3]. In the Premier Healthcare Database study, the 90-day mortality after a pathological fracture was about 10% [2].

One of the common treatments for bone metastases is focal external beam radiation therapy (EBRT). Other common treatments are chemotherapy, biological therapies, bisphosphonates, RANK-L inhibitors, and radionuclides. Before these effective systemic therapies became available, systemic external beam radiotherapy was given by using an approach called hemibody irradiation (HBI). HBI involves the treatment of half of the entire body (or skeletal system) in a single field of radiation therapy. This was most often given as upper hemibody irradiation (UHBI) and lower hemibody irradiation (LHBI) or sequential UHBI and LHBI.

The first report of HBI for bone metastases was published by Fitzpatrick and Rider based on a study at the Princess Margaret Hospital in 1976, after presenting it at the American Society of Therapeutic Radiation Oncology’s annual meeting in 1974 [4]. They were aware that a palliative dose of whole-body radiation therapy sufficient to treat bone metastases would be lethal but hypothesized that by treating one half of the body at a time the unirradiated marrow would subsequently repopulate the treated, ablated marrow. They reported their procedure as follows:

“Half-body irradiation requires special physical parameters. These include large fields up to 120cm^2^ and a high output which will deliver a tumor dose of 1000 rad (1 rad = 1 cGy) in a short time. We accomplished this by using either an Eldorado Co-60 unit equipped with a 9000 Ci source or a Clinac 35 MV Linear Accelerator. Source-to-skin distances of up to 200 cm were used, and shields, bolus, and compensators delineated field size and homogenized the radiation dose. The tumor dose was measured at the midplane; half the irradiation was delivered through an anterior field and half through a posterior field. Tumor doses of 500, 600, 800, and 1000 rad, uncorrected for specific tissue absorption, were delivered in a single exposure. The radiation time varied from 5 to 30 min with a total treatment time of 30-60 min. Because these treatments were palliative, some patients received only partial half-body radiation; for example, metastases in breast cancer are uncommon below the knees, so treatment was not given to the lower leg when there was no evidence of disease. Similarly, we shielded the eyes and omitted the head above the skull base when disease was absent, because of the associated morbidity from epilation of the scalp.” [4].

They presented the results of 82 patients treated from June 1971 to March 1974, the majority with breast cancer (n=58). LHBI was well tolerated, but UHBI caused moderately severe retching, nausea, vomiting, and diarrhea. These symptoms started one hour after treatment and generally lasted for several hours but could last for days. A week after radiation therapy some patients developed mucositis or vulvitis. After LHBI or UHBI, the platelets and hemoglobin did not decrease significantly, and the white cell counts fell slightly 10-21 days after treatment and recovered 28-35 days after treatment. No patient developed kidney failure. The limiting toxicity was pneumonitis, and several patients who underwent 800 or 1000 cGy UHBI died from pneumonitis. They did not provide response rates but stated that there was a dramatic reduction in pain within 24 hours of treatment.

Subsequent research led to the standardization of the treatment to 800 cGy LHBI and 600 cGy UHBI. A lung density correction (10% increased transmission) was added by most investigators, and shielding was sometimes added to the UHBI to reduce the dose to the kidneys and lungs [5]. The matching point for the two fields was generally placed at the iliac crests or the bottom of L4. The feet and skull were variably included. Premedication regimens were given, often including hospitalization for hydration, corticosteroids, and an antiemetic such as prochlorperazine [6]. More recent papers have reported HBI given to only the bones using 3D planning, Tomotherapy®, and volumetric arc therapy [7-9]. 

If further research on HBI using modern treatment techniques confirms that it is effective and safe, it could be a cost-effective and efficient approach to treat bone-only metastatic disease in prostate, breast, and other cancers. In light of this, we performed this systematic review and meta-analysis to determine the efficacy and tolerability of HBI.

## Review

Methods

This meta-analysis involves primary reports on the treatment of pain in patients with bone metastases from solid tumors and treated with single-fraction HBI therapy. PRISMA guidelines were followed in performing this study [10]. Papers were found based on a PubMed search using the phrase “(radiotherapy AND (halfbody OR hemibody OR half-body OR hemi-body))” and limited to humans, with no limitation on language and date. The abstracts were then reviewed (or full papers if no abstract was available) and an article was selected if it fulfilled the meta-analysis focus. Further evaluation eliminated papers that did not involve treatment with a single fraction or were redundant reports of a single database. The remaining papers were reviewed for further relevant references. The papers were also evaluated through Scite_® for further potential references based on references back to the selected paper.

The relevant data were extracted from the papers. Due to limitations in the quality of the reports, the primary endpoint was confined to overall pain response, as defined within the paper being reviewed. Complete rates were also recorded, if available. In several papers, response per treatment rather than response per patient was given, with several patients receiving both upper and lower hemibody treatment [11-13]. In those cases, it was assumed that the response rate for upper and lower hemibody treatments was equal, and the proportion of responses per treatment was translated to be the response per patient by multiplying the number of patients by the overall response rate. This was done to allow conformity in the reporting of the results between the studies. The overall quality of the studies was poor by modern standards. Therefore, only a crude quality factor was applied: retrospective studies were given a quality factor of 0.33, prospective studies (including Phase I and II) were rated 0.66, and randomized trials were rated 1.0.

A single-factor proportional meta-analysis was performed. This was done using MetaXL® (Version 5.3, available at https://www.epigear.com/index_files/metaxl.html) on Microsoft Excel® software. This analysis is based on a double arcsine transformation, which is the preferred method over logit transformation [14]. This approach is not uniformly accepted, but for this study, a more complex analysis, such as a Bayesian model, would not increase the veracity of the data [15]. Because of the wide variability and poor definitions of response, a random rather than fixed model was used for the primary analysis. Comparisons of the random model with the results of a fixed model as well as a heterogeneity-corrected model were also performed.

Results

**Figure 1:**
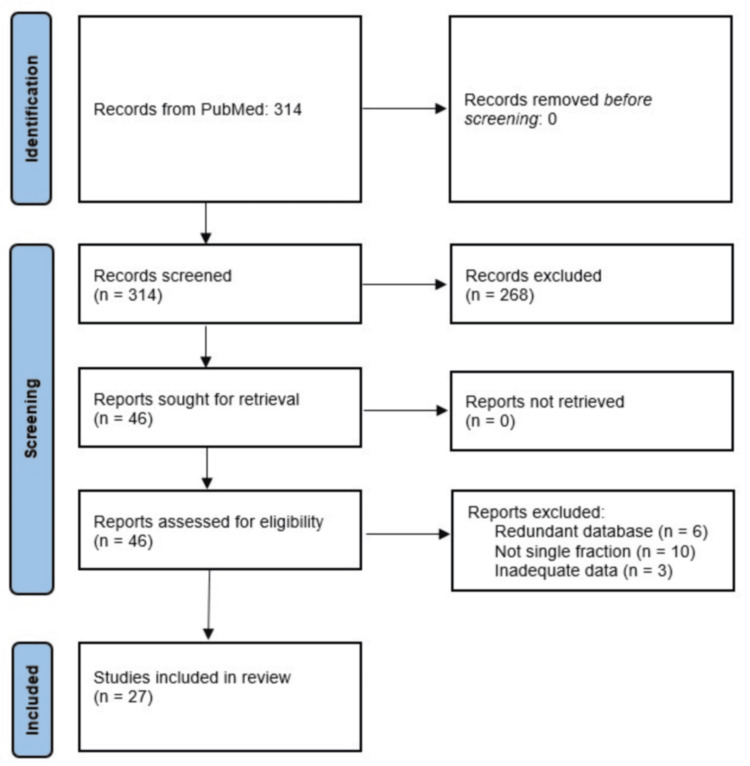
PRISMA flow diagram depicting the selection of studies PRISMA: Preferred Reporting Items for Systematic Reviews and Meta-Analysis

The primary PubMed search yielded 314 references. The articles’ references and the Scite_® searches added no new papers. All 314 references were screened for relevance, and 46 articles that focused on patients with bone metastases from solid tumors were selected. Redundant articles on the same databases (n=6), articles without data (n=3), and non-single fraction trials (n=10) were then eliminated. This left us with 27 primary reports involving 1318 patients suitable to be analyzed. These references are listed in Table 1. These reports span a 44-year period. Figure 1 shows the Preferred Reporting Items for Systematic Reviews and Meta-Analysis (PRISMA) flow diagram illustrating the selection of studies.

**Table 1:** Studies included in the meta-analysis No. Pts: number of patients; OR: overall response rate; CR: complete response rate

Study	Year	No. Pts	OR	CR
Salazar et al. [20]	1978	25	21	12
Epstein et al. [21]	1979	10	8	
Keen [22]	1980	49	33	15
Mill et al. [23]	1980	17	15	2
Qasim [24]	1981	92	70	46
Rowland et al. [25]	1981	52	42	
Algara and Valls [26]	1984	71	65	27
Pouisson-Rosillo et al. [27]	1985	32	24	
Salazar et al. [16]	1986	129	94	25
Itami et al. [28]	1987	7	5	1
Wilkins and Keen [17]	1987	92	75	15
Reed et al. [29]	1988	50	39	
Hoskin et al. [12]	1989	30	27	
Nseyo et al. [13]	1989	19	19	
Burmeister and Probert [30]	1990	16	15	
Zajic et al. [31]	1990	13	11	
Dearnaley et al. [32]	1992	27	17	
Chua et al. [33]	1994	123	105	
Quilty et al. [34]	1996	46	32	
Salazar et al. [35]	1996	74	54	
Skolyszewski et al. [18]	2001	102	77	25
Biswal [36]	2004	50	50	15
Bashir et al. [19]	2008	103	76	
Berg et al. [5]	2009	34	26	3
Furlan et al. [8]	2014	13	11	8
Pal et al. [37]	2014	23	20	5
Kluska et al. [9]	2022	19	16	6

Upper and lower hemibody treatments had similar response rates across all studies that reported these outcomes [5,9,16-19]. This justified the data conversion from response rate per treatment to response rate per patient, enabling direct comparisons between the trials. The distribution of the types of cancer treated are shown in Table 2. Not all papers documented the types of cancer treated. As shown in Table 2, half of the patients with reported sites had prostate cancer and a quarter had breast cancer.

**Table 2:** Types of cancers treated

Type of cancer	Number of patients, (%)
Prostate	562 (49%)
Breast	286 (25%)
Lung	103 (9%)
Other	202 (17%)

**Figure 2:**
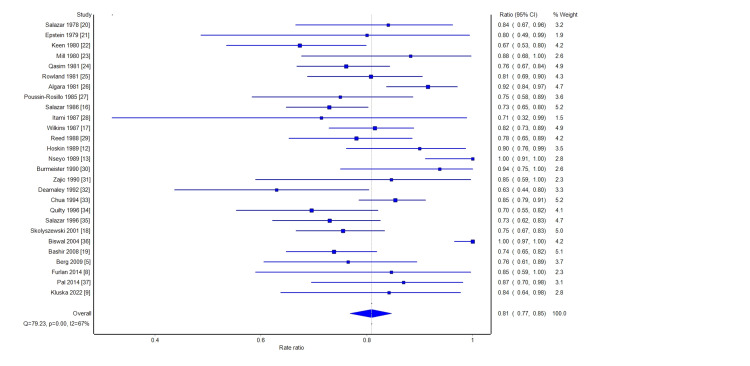
Forest plot depicting the overall response rate CI: confidence interval

**Figure 3:**
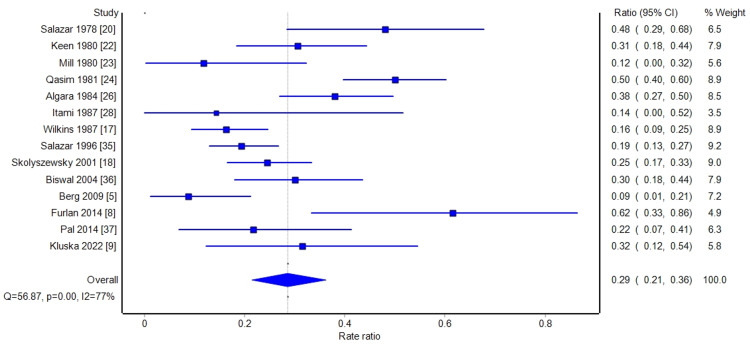
Forest plot depicting the complete response rate CI: confidence interval

**Figure 4:**
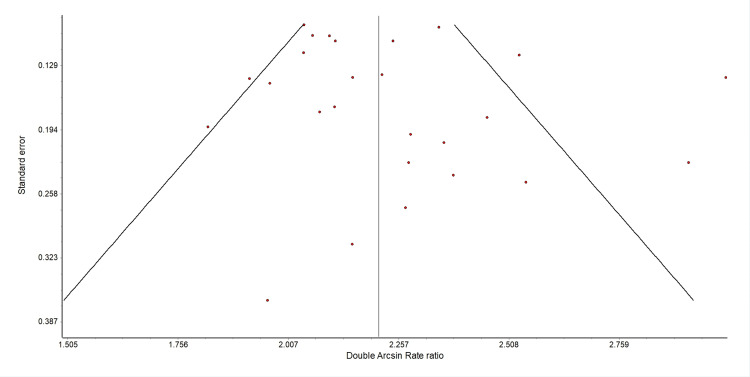
Funnel plot depicting the overall response rate

**Figure 5:**
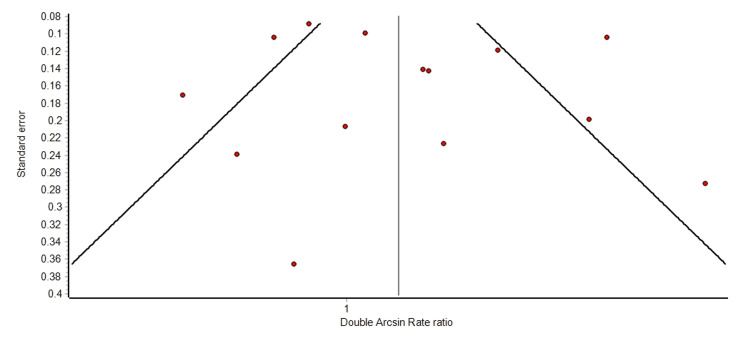
Funnel plot depicting the complete response rate

A meta-analysis was performed on the response rates (combined partial and complete) to hemibody radiation therapy. A forest plot of the overall response rates is shown in Figure 2. The summary analyses are shown in Table 3. Per the random effect meta-analysis, 80% (95% CI: 76% to 84%) of patients had a complete or partial pain response with HBI. The same proportion of responses was found in fixed result analysis and with quality-adjusted analysis. Among those papers that reported complete response rates, on meta-analysis, 29% of patients had a complete response (95% CI: 22% to 37%). These results are shown in Figure 3 and Table 4. The heterogeneity between trials, based on the Q statistic and the l^2^ statistic, was large. This was expected due to the heterogeneity in reporting the results and definition of endpoints. However, as shown in the overall response rate and complete response rate funnel graphs (Figures 4-5), there was no significant bias in the distribution of the results.

**Table 3:** Meta-analysis of overall response rate CI: confidence interval

Method	Prevalence	Lower CI	Upper CI	
Fixed effects	0.7992	0.7773	0.8203	
Random effects	0.8092	0.7681	0.8473	
Quality effects	0.8023	0.7571	0.8440	
Fixed effects, heterogeneity	0.7992	0.7540	0.8423	

**Table 4:** Meta-analysis of complete response rate CI: confidence interval

Method	Prevalence	Lower CI	Upper CI
Fixed effects	0.2795	0.2475	0.3126
Random effects	0.2856	0.2145	0.3624
Quality effects	0.2856	0.2112	0.3662
Fixed effects, heterogeneity	0.2795	0.1994	0.3638

Discussion

This meta-analysis is limited by the quality of the data gathered. Many of these studies are from 30 to 50 years ago, and among the studies, there was no standardization of reporting, no standard approach to defining a response, and there were very few prospective studies about the technique. Although half of the patients had prostate cancer, a quarter of the patients had breast cancer and another quarter had other cancers. Therefore, no matter how precise a statistical analysis is performed, the underlying level of the data quality is poor. However, as shown in the funnel plots of the data (Figures 4-5), there does not appear to be significant asymmetry suggesting report bias. Hence, it is reasonable to discuss the implications of these results while still acknowledging their limitations.

The overall response rate for pain relief was 80%, with a tight CI range of 77% to 85%. The complete response rate, when reported, was about 30% (calculated as 29%, 95% CI: 22% to 37%). In a contemporaneous trial by the Radiation Therapy Oncology Group (RTOG) from the latter half of the 1970s, patients were randomized to different regimens of focal external beam radiation therapy for the palliation of pain from bone metastases [38]. Overall, 83% of patients had complete or partial pain relief and 53% had complete pain relief. In a more recent study by the NRG-Oncology Research Group (the successor to RTOG) with more rigorous pain measurement, patients receiving 800 cGy in one fraction to bone metastases had a 61% complete or partial pain response [39]. Thus, the results of the meta-analysis are in line with the results from focal EBRT.

Historically, the volume of the radiation field was generally limited to the site of metastasis to minimize toxicity, especially marrow toxicity. An important factor that contributed to the introduction of HBI was previous work done at the Princess Margaret Hospital in Toronto [40]. Radiobiological studies, both cell culture and spleen colony assays, showed 300 cGy to have a hemopoietic cell lethality of 90% (followed by full recovery) and 99.9% lethality at 800 cGy with no recovery [41,42]. These results suggested that only 10% of marrow was required for recovery. They hypothesized that, given the appropriate interval, the unirradiated bone marrow would circulate and repopulate the ablated marrow. The clinical experience with “half” body radiation (hemibody) by Fitzpatrick and Rider for palliation of pain from multiple sites confirmed the feasibility of HBI [4]. The initial report involved 82 patients, and all received 800 cGy upper and lower HBI. They stated that lower HBI led to few or no side effects while upper HBI was followed by nausea, vomiting, diarrhea, and pneumonitis.

The first report from the United States was published in 1978, based on a study at the Strong Memorial Hospital in Rochester, NY [20]. All patients received a single dose of 800 cGy at a low dose rate, with fields that included the upper half body (including head to pelvic brim) in 40 patients and the lower HBI (pelvic hemibody to below the feet) in 23 patients. In general, the main clinical toxicities associated with upper HBI, when minimal premeds were utilized, were moderate to severe nausea, vomiting, chills (acute radiation syndrome), an increase in temperature and pulse rate, and a drop in blood pressure. The onset occurred at the completion of HBI. Subsequently, the final six patients all received antiemetics, adequate hydration, and corticosteroids and this significantly reduced the toxicity. The limiting severe toxicity was the occurrence of radiation pneumonitis after upper HBI, which was associated with 800 cGy doses (uncorrected for lung transmission). The authors suggested that future treatments should use a dose of 600-700 cGy corrected for lung transmission. Also, aggressive pretreatments, such as hydration, prophylactic anti-emetic, and prophylactic corticosteroid use, were instituted to reduce toxicity and improve the tolerance of HBI.

The final analysis of the RTOG protocol 78-10, published in 1986, reported the results of a prospective trial employing increasing single doses of HBI in patients with multiple osseous metastases [16]. Several different fractionation schemes were employed. Upper HBI received 600 and 700 cGy while mid and lower HBI received 800, 900, and 1000 cGy. Pain relief was noted in 73% of patients following HBI and 80% had pain relief within one week. Toxicity following mid and lower HBI primarily involved mild to moderate nausea, vomiting, and diarrhea in 10% of patients. Toxicity following upper HBI was primarily nausea and vomiting, which was rated as severe in 16% of patients. Severe and life-threatening hematologic toxicity was observed in one-third of 40 patients receiving upper HBI, primarily in individuals who had received prior chemotherapy and had very low baseline counts at the time of HBI.

Berg et al. reported a smaller prospective study of 44 male and one female patient with metastatic disease in 2009 [5]. Forty-one patients had prostate cancer, one had lung cancer, one had an unknown primary, and the female patient had breast cancer. Thirty-seven patients received lower HBI, five were treated with upper hemibody, and two had both upper and lower HBI. The study is notable because it reported on the patients over a 24-week period following HBI. The patients were seen at weeks 2, 4, 8, 16, and 24 following HBI. Vomiting occurred in 42% of patients at baseline and increased to 50% at two weeks but then decreased to about 25% over the ensuing weeks. Diarrhea occurred in 49% of patients within two weeks of HBI and then decreased to 13% of patients. All cases of diarrhea occurred in patients receiving lower HBI and none in the five patients receiving upper HBI. Fatigue was present in 40% of all patients on presentation, increasing to 63% at week two, and then decreased at eight weeks but after that rose again, up to 60%. Mild pulmonary symptoms (graded as 1-2) were reported in those patients receiving upper HBI.

Modern techniques can decrease toxicity while maintaining efficacy. Furlan et al. reported on the use of high-precision hemibody treatment using helical tomotherapy on 13 breast cancer patients with painful metastases in the lower hemibody [8]. All patients received a single dose of 800 cGy to the lower hemibody with the target limited to the bones. They reported that 85% of patients had pain relief, of which 62% had complete pain relief. Overall toxicity was acceptable: two grade 3 hematologic toxicities while the rest, including fever, nausea, and diarrhea, were all grades 1 or 2. Macchia et al. used 3D-conformal HBI focused on the bones to reduce the potential toxicity of the treatment [7]. The HBI dose-fractionation was 300 cGy twice a day for two days, for a total dose of 1200 cGy. They treated the pelvic bones, lumbar-sacral vertebrae, and upper third of femurs. The pain response rate was 76% with a 38% complete response rate. They reported grade 3 and 4 acute toxicity rates of 1% and 0% among the 180 patients treated. The hematologic grade 3 toxicity rate was 0.6%, with a 0% grade 4 rate.

Zamagni et al. conducted a phase 1 study that treated patients with increasing doses, from 225 cGy to 375 cGy, twice a day for two days, to a field covering the lumbar vertebrae to the femurs (upper third or whole femur depending on disease extent) [43]. Twenty-five patients were treated. The target volume was limited to the bones. The overall pain response rate was 76% with a 36% complete response rate. There was no grade 3 or 4 toxicity. Kluska et al. performed a retrospective analysis of hemibody radiation done with VMAT arc therapy [9]. They treated 22 patients with either or both 600 cGy upper HBI or 800 cGy lower HBI. The target was limited to the bones, with target mean doses of less than 250 cGy for the kidneys, 300 cGy for the rectum, bladder, and heart, and 400 cGy for the liver and the lungs. They had an 80% overall pain reduction rate with a 10% complete response rate. They reported that only one patient had a grade 3 toxicity (anemia requiring transfusion). No study using bone-targeted planning has reported an incidence of death [7-9,43]. These studies show that the initially reported high toxicity of the HBI can be reduced to acceptable levels with modern, more advanced radiation therapy planning and delivery techniques, without compromising on the response rates.

Alternative dosing approaches, such as fractionation, may also help to reduce the risk of toxicity. For example, Salazar et al.'s 2001 phase III international trial of fractionated HBI randomized 146 patients with symptomatic bone metastases to upper or lower hemibody in dose regimens of 1500 cGy in five fractions over three days, 800 cGy in two fractions in one day, or 1200 cGy in four fractions over two days [44]. No advanced planning techniques were used in this trial. Nearly 40% of patients had no treatment complications, about 50% had mild or moderate toxicity, and 12% had severe toxicity. The authors reported no treatment-related deaths. The primary upper hemibody toxicities were nausea and vomiting and hematologic, while the lower hemibody toxicities primarily involved diarrhea and hematologic. Of note, 91% of the patients had some level of pain relief and 45% had complete pain relief.

Fatal toxicity from HBI is rare. Among the 1318 patients reported in this analysis, the death rate due to treatment was 2.1% (28 patients). Most of these deaths were due to radiation pneumonitis and were found in very early studies that gave 800 cGy upper hemibody treatment, rather than the later standard 600 cGy dose. If the patients receiving 800 cGy or higher UHBI are discounted, the rate of fatal toxicity comes down to 0.6% (eight patients). These include one pulmonary toxicity with 600 cGy (Hoskin et al., 1989) [12], one due to thrombocytopenia (Mill et al., 1980) [23], two due to marrow failure (Rowland et al., 1981) [25], two due to enteritis (Algara et al., 1994) [11], and two myocardial infarctions due to acute radiation syndrome with hypotension (Salazar et al., 1978) [20]. There were no deaths in Salazar et al.'s subsequent reports (1986, 1996) [16,35].

HBI was never a mainstream treatment and is rarely used today. The current whole-body radiotherapy approach for treating bone metastases involves the use of radiopharmaceuticals. Unlike external beam radiotherapy, radiopharmaceuticals are delivered intravenously, analogous to chemotherapy or biologically targeted therapy. The radiation is delivered directly to the target cancer cells or the tumor microenvironment. Radiopharmaceuticals used for treating bone metastases include the radionuclides P-32, Sr-89, Re-186, Re-188, Sm-155, Ra-223, and 177Lu-PSMA-617, These agents, except 177Lu-PSMA-617, function by being taken up in the active bone around the metastases, either as a calcium analog or as a phosphate ion. Lu177-PSMA-617 binds directly to receptors on the prostate cancer cells. These agents differ in treatment efficacy, duration of pain palliation of symptoms, tumoricidal effects, toxicity, number of treatments, and financial burden. Most studies with radiopharmaceutical agents have been conducted in prostate and breast cancer patients.

A systematic review of the use of radiopharmaceuticals for the palliation of bone pain from metastases showed that the pain relief from Sr-89 was in the range of 50-60% and that from Sm-153 was in the 70% range [45]. The primary toxicity with radionuclides was hematological suppression. The pain response to a series of Ra-223 treatments with 100 kBq/kg was in the 50-60% range [45]. There is sparse literature on per-patient pain relief from Lu-177-PSMA-617 treatment, with one paper reporting that 10 of 27 patients (37%) had a reduction in pain by the second round of treatment [46]. Two papers compared HBI with Sr-89 therapy. Dearnaley et al. [32] retrospectively compared matched patients, 27 receiving HBI at the Royal Marsden Hospital vs. 51 patients receiving Sr-89 at the Southampton General Hospital. Pain response was seen in 63% of the HBI patients and 52% of the Sr-89 patients. There was no difference in survival between the matched patients. Quilty et al. performed a randomized trial comparing HBI and Sr-89 treatment [34]. The pain relief at three months was 66% after Sr-89 and 61% after HBI. There was no significant difference in survival. WHO grade III/IV platelet toxicity was evident in 11 patients after strontium-89 (6.9%), compared with five patients after radiotherapy (3.4%). HBI led to significantly more nausea, vomiting, and diarrhea (43% vs. 10%). Overall, the present meta-analysis data appear to show that the ability of HBI to palliate pain is equivalent to that of radiopharmaceuticals.

Advantages in terms of survival with treatment have been seen with modern radiopharmaceuticals in prostate cancer patients. Ra-223 was evaluated in a double-blind, randomized, multiple-dose, phase III multicenter study (ALSYMPCA) in castration-resistant prostate cancer patients with bone metastases [47-49]. The results showed that the overall median survival improved in patients treated with Ra-223 plus best standard of care compared to patients treated with placebo plus best standard of care: the median survival increased to 14.9 months from 11.3 months. The study also showed a significant increase in time to the first skeletal-related event, defined as time to the need for EBRT, time to first pathological bone fracture, time to spinal cord compression, or time to surgical intervention. The phase III VISION trial evaluated the addition of 177Lu-PSMA-617 to best supportive care on the overall survival and image-based progression-free survival in patients with progressive metastatic castration-resistant prostate cancer [50]. All patients had a positive gallium-68-labeled PSMA-11 PET scan. There was a statistically significant four-month improvement in the median overall survival of patients who received 177Lu-PSMA-617 over standard care (15.3 months vs. 11.3 months). There was a higher incidence of adverse effects in the intervention 177Lu-PSMA-617 arm (52.7% vs. 38%) but the quality of life remained the same.

To date, no study of HBI has been designed to show a survival advantage. However, Salazar et al.'s randomized trial in 2001 has generated suggestive data [44]. In that trial, previously discussed above in the context of toxicity, the three arms were as follows: 1500 cGy in five fractions over three days (Arm A), 800 cGy in two fractions in one day (Arm B), and 1200 cGy in four fractions over two days (Arm C). The majority of the patients had breast or prostate cancer. The tumor-related 200 cGy equivalent doses (EQD2) quoted in the paper were 1960, 880, and 1620 cGy for arms A, B, and C, respectively (the formula used for this calculation was not stated). Thus, Arm B received a 50% lower equivalent dose than Arms A and C. The mean survival times for these advanced cancer patients were 175 days (Arm A), 155 days (Arm C), and 104 days (Arm B). This two-month gain in survival for A and C vs. B was statistically significant (p=0.042). This suggests a possible survival advantage for higher-dose HBI. Another important feature of this trial was that it was an international trial open from 1996 to 1999 in countries including Brazil, Cameroon, Pakistan, and Peru, showing that this technique is easily transferrable to less medically developed countries.

In the United States, the cost of a standard course of six doses of Ra-223 (Xofigo®) is estimated to be $180,000 [51], and a standard course of six doses of 177Lu-PMSA-617 (Pluvicto®) is estimated to cost $270,000 [52]. Due to the high costs of Ra-223 and Lu-177-PSMA-617 and the small survival advantage of only three to four months, these agents are not considered to be cost-effective treatments [53-56]. In comparison, a single fraction of external beam IMRT radiation therapy, including simulation, planning, and delivery, using standard Medicare reimbursement, costs approximately $3200. Therefore, a course of sequential upper and lower hemibody external beam treatments would be about $6400 for a two-section HBI or $9600 for a three-section hemibody treatment. This raises the possibility that HBI could prove to be a more cost-effective approach to treating patients with widespread bone metastases.

There is a wide disparity in cancer mortality between developed countries and less developed countries. The age-standardized incidence of cancer among men in very high human development index (HDI) countries is 335.3 per 100,000 compared to 104.3 in low HDI countries [57]. However, the age-standardized mortality rates from cancer are significantly closer: 122.9 in very high HDI countries vs. 78.0 in low HDI countries. The statistics for women are more dire. The incidence among women in very high HDI countries is 267.6 compared to 128.0 in low HDI countries. However, the risk of mortality from cancer is higher in low HDI countries than in very high HDI countries: 88.4 vs. 80.0. These profound health disparities should drive the very high HDI countries to develop effective treatment methods transferable to low HDI countries [58]. HBI may be a universal approach to systemic radiotherapy for patients with bone metastases.

## Conclusions

Although the concept of HBI is foreign to modern radiation oncologists, research conducted over the last 50 years has shown it to be efficacious for pain relief from bone metastases. Recent studies using modern radiation therapy techniques that limit the target to the bones, rather than treating the body through and through, have shown that it can be delivered with minimal grade 3 or higher toxicities. This review indicates that HBI has palliative effects for pain from bone metastases equivalent to those of focal EBRT and radiopharmaceuticals. Therefore, the modern HBI method has the potential to both cheaply and effectively palliate pain due to widespread bone metastases. Thus, it is important to prospectively evaluate the ability of HBI using modern techniques to safely relieve pain and possibly to improve survival. We recommend further studies on HBI using modern radiation therapy planning and delivery techniques.
